# Prevalence and risk distribution of schistosomiasis among adults in Madagascar: a cross-sectional study

**DOI:** 10.1186/s40249-023-01094-z

**Published:** 2023-04-25

**Authors:** Sarah Katharina Gruninger, Tahinamandranto Rasamoelina, Rivo Andry Rakotoarivelo, Anjarasoa Ravo Razafindrakoto, Zaraniaina Tahiry Rasolojaona, Rodson Morin Rakotozafy, Patrick Richard Soloniaina, Njary Rakotozandrindrainy, Pia Rausche, Cheick Oumar Doumbia, Anna Jaeger, Alexandre Zerbo, Heidrun von Thien, Philipp Klein, Govert van Dam, Egbert Tannich, Norbert Georg Schwarz, Eva Lorenz, Jürgen May, Raphael Rakotozandrindrainy, Daniela Fusco

**Affiliations:** 1 Department of Infectious Disease Epidemiology, Bernhard Nocht Institute for Tropical Medicine, Hamburg, Germany; 2 German Centre for Infection Research (DZIF), Hamburg-Borstel-Lübeck-Riems, Germany; 3 Centre d’Infectiologie Charles Mérieux (CICM), University of Antananarivo, 101 Antananarivo, Madagascar; 4 Department of Infectious Diseases, University of Fianarantsoa Andrainjato, 301 Fianarantsoa, Madagascar; 5 Department of Microbiology and Parasitology, University of Antananarivo, 101 Antananarivo, Madagascar; 6 University Clinical Research Centre (UCRC), University of Sciences Technics and Technologies of Bamako (USTTB), Bamako, Mali; 7 Department of Parasitology, Leiden University Medical Centre, Leiden, The Netherlands; 8 National Reference Centre for Tropical Pathogens (NRC), Hamburg, Germany; 9 Institute of Medical Biostatistics, Epidemiology and Informatics, University Medical Centre of the Johannes Gutenberg University Mainz, Mainz, Germany; 10 Department of Tropical Medicine I, University Medical Center Hamburg-Eppendorf (UKE), Hamburg, Germany

**Keywords:** Schistosomiasis, *Schistosoma haematobium*, *Schistosoma mansoni*, Universal health coverage, Madagascar

## Abstract

**Background:**

The goal to eliminate the parasitic disease of poverty schistosomiasis as a public health problem is aligned with the 2030 United Nations agenda for sustainable development goals, including universal health coverage (UHC). Current control strategies focus on school-aged children, systematically neglecting adults. We aimed at providing evidence for the need of shifting the paradigm of schistosomiasis control programs from targeted to generalized approaches as key element for both the elimination of schistosomiasis as a public health problem and the promotion of UHC.

**Methods:**

In a cross-sectional study performed between March 2020 and January 2021 at three primary health care centers in Andina, Tsiroanomandidy and Ankazomborona in Madagascar, we determined prevalence and risk factors for schistosomiasis by a semi-quantitative PCR assay from specimens collected from 1482 adult participants. Univariable and multivariable logistic regression were performed to evaluate odd ratios.

**Results:**

The highest prevalence of *S. mansoni, S. haematobium* and co-infection of both species was 59.5%, 61.3% and 3.3%, in Andina and Ankazomborona respectively*.* Higher prevalence was observed among males (52.4%) and main contributors to the family income (68.1%). Not working as a farmer and higher age were found to be protective factors for infection.

**Conclusions:**

Our findings provide evidence that adults are a high-risk group for schistosomiasis. Our data suggests that, for ensuring basic health as a human right, current public health strategies for schistosomiasis prevention and control need to be re-addressed towards more context specific, holistic and integrated approaches.

**Supplementary Information:**

The online version contains supplementary material available at 10.1186/s40249-023-01094-z.

## Background

Access to health care is a human right and the urge of achieving universal health coverage (UHC) is emphasized by the United Nations (UN) in the sustainable development goals (SDGs) [1, 2]. The control of infectious diseases of poverty, and particularly those characterized by long term consequences, have been suggested as indicators towards UHC [3–5].

Schistosomiasis is a waterborne neglected disease of poverty prevalent in tropical areas but not limited to them [6]. It is caused by different species of the trematode schistosome, of which *S. mansoni* and *S. haematobium* are the most frequent worldwide [7]. The Neglected Tropical Diseases (NTDs) roadmap, released by the World Health Organization (WHO) in 2021 [8], targets the elimination of the disease as a public health problem by 2030 in all endemic countries. Progress on morbidity control has been made through vertical control strategies, especially by preventive chemotherapy through mass drug administration (MDA) with praziquantel in school aged children [9–11]. However, it has been reported that the implementation of vertical control programs can exacerbate health inequalities amplifying disparities based on sex, gender, socio-economic status and age [3, 12–14], hampering the achievement of UHC. Even though adults in high-risk areas and with occupational risk are theoretically eligible to receive annual MDA [15], campaigns including adults are rare [16]. In 2019, the WHO reported a global coverage rate of 67.2% in school aged children, while it was only 17.7% in adults [17]. The limited availability of praziquantel on the market alongside with the low MDA uptake of adults because of occupational duties, fear of side effects and understanding schistosomiasis as a pediatric disease, exclude de facto adults from national programs [16]. As a consequence, untreated adults do not only serve as infection reservoir for the community, but are also under risk to develop severe chronic forms of the disease [16].

Chronic intestinal schistosomiasis caused by *S. mansoni* can lead to hepato-splenomegaly and portal hypertension, chronic urogenital schistosomiasis caused by *S. haematobium* carries high risk of squamous bladder cancer and manifestations like genital schistosomiasis [7]. Both forms may present symptoms like chronic pain, fatigue and morbidities like anemia and undernutrition, resulting in a great loss of quality-adjusted life-years (QALY) [18, 19], impaired work capacity of adults [20] and finally reduced economic productivity [7]. Adapted guidelines for the diagnosis and treatment of chronic forms of schistosomiasis are often missing in endemic countries generating a lack of services aggravated by the low knowledge among the health-care workers of signs, symptoms and management of chronic forms of the disease, such as female genital schistosomiasis [8, 21]. Preventive chemotherapy at early stages of life, early diagnosis and treatment can prevent long-term consequences and increase QALY [22]. Diagnostic services are still limited in low resource settings because of a lack of affordable and easy to implement diagnostic tools [23]. Moreover, the health seeking behaviors for schistosomiasis care show several barriers as for example social stigma or opening hours of health services that often overlap with the working hours of the users limiting their accessibility [24, 25]. Even though treatment of chronic forms in adults might be available, it can be linked to out-of-pocket expenses as health interventions and transport costs to the health care centers are frequently not affordable for the affected populations [26]. Further, patients, especially women, with morbidities of chronic genital schistosomiasis, like infertility and vaginal discharge, fear marginalization when accessing health services as the symptoms resemble those of sexually transmitted diseases [27].

The life cycle of schistosome infection [7] justifies the adoption of a holistic approach combining MDA with health education programs and improved water, sanitation and hygiene (WASH) [28] measures in a One Health-oriented [29] approach to tackle the disease at its sources [8, 10]. Infection control measures should be encouraged in low resource settings to control morbidity, prevent chronic forms of the disease and meet the ambitious goal of the NTD roadmap [8]. However, several infection control interventions have so far proven to be ineffective for schistosomiasis prevention and control due to contextual barriers that demand the synergistic implementation of multiple measures in order to impact on the transmission of the disease [7, 30].

This study is based on data collected in Madagascar, a country highly endemic for schistosomiasis where both, *S. mansoni* and *S. haematobium* exist in distinct geographic areas [31, 32]. With the exception of few surveys conducted among adults the most of the existing data in the country refers to school-aged children with prevalence ranging between 15.2% and 87.7% [33–36].

Our study aims to provide prevalence estimates, describe risk associations of schistosomiasis in adults in the country and motivate a shift in the paradigm of schistosomiasis control programs from targeted to broader approaches as key element for both the elimination of schistosomiasis as a public health problem and the promotion of UHC.

## Materials and methods

### Study design, area and population

The planning, organization, implementation and analysis of this cross-sectional study was conducted by eight interdisciplinary collaborating institutes: the Bernhard Nocht Institute for Tropical Medicine, Hamburg, Germany; the German Centre for Infection Research, Hamburg-Borstel-Lübeck-Riems, Germany; the *Centre d’Infectiologie Charles Mérieux*, University of Antananarivo, Madagascar; University of Fianarantsoa Andrainjato, Madagascar; University Clinical Research Centre, Bamako, Mali; Leiden University Medical Centre, Netherlands; Johannes Gutenberg University Mainz, Germany.

**Fig. 1:**
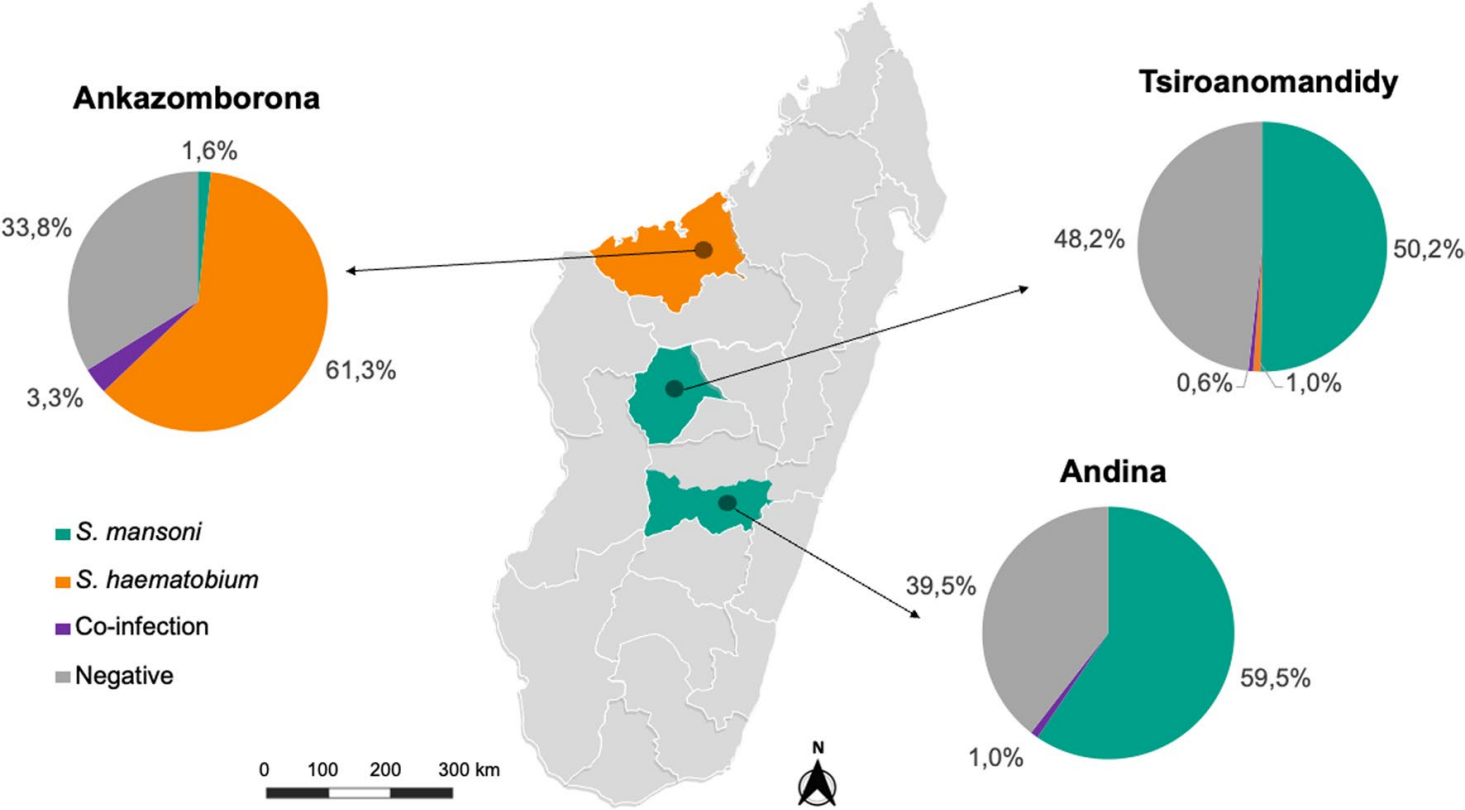
Map of Madagascar showing the crude prevalence of *Schistosoma* species and co-infection status based on PCR at the study sites, *n* = 1482. Map was adapted using a template from https://yourfreetemplates.com

This study was conducted at three Primary Health Care Centers (PHCCs) in Madagascar: Andina (20°30′58.8″S 47°09′05.2″E) and Tsiroanomandidy (18°46′21″S 46°02′57″E) in the centre, Ankazomborona (16°06′50″S 46°45′24″E) in the north western part of the island (Fig. 1). On the basis of the infrastructure and population size, Andina can be described as rural area, Ankazomborona and Tsiroanomandidy as peri-urban and urban areas, respectively [37], even though this classification cannot be aligned to the standard definition of the UN statistical commission due to the limited available data of the study sites [38].

Information sessions about the study were organized in the catchment areas of the included PHCCs. If interested, individuals were asked to attend PHCCs to assess their eligibility for the study (18 or older, not pregnant and willing to comply with protocol requirements). If eligible, an informed consent was signed in case of enrolment.

### Data collection and management

Data collection took place between March 2020 and January 2021. Background characteristics of the participant were collected by means of a case report form (CRF). Completed CRFs were checked for missing entries manually by local study nurses and doctors following standard operating procedures. CRF data were fed into the database REDCap^®^ (Vanderbilt University, Nashville, USA) via double data entry by two independent operators. Quality control of data processing and data validation was undertaken at regular intervals during the course of data entry.

### Sample collection and management

For this study a sample of 9 ml of venous blood was collected from each participant. Blood samples were centrifuged at 1600 × g for 10 min and two aliquots of 1 ml each of serum was produced by qualified laboratory technicians. Samples were stored according to required quality standards at – 20 °C and transferred to long-term storage at – 80 °C in Madagascar. At the end of sample collection, one of the serum aliquots was shipped to Hamburg on dry ice and stored at – 80 °C until the analysis was performed.

### Sample analysis

DNA was extracted with QIAamp MinElute ccfDNA Mini Kit from 1 ml serum according to the manufacturer’s instructions (Qiagen, Hilden, Germany). Extracted DNA was stored at – 20 ℃.

The semi quantitative standardized polymerase-chain reaction (qPCR) analysis was based on the previously published protocol by Frickmann et al. [39] with described sensitivity of 94.9% and specificity of 98.4%. The primers used for the amplification were: *S. mansoni*—Forward Primer: 5′ CAA CCG TTC TAT GAA AAT CGT TGT 3′, *S*. *mansoni*—Reverse Primer: 5′ CCA CGC TCT CGC AAA TAA TCT 3′, *S*. *mansoni*—Probe: ‘Fam-TCC GAA ACC ACT GGA CGG ATT TTT ATG AT-BHQ1′, *S*. *haematobium*—Forward Primer: 5′ GAT CTC ACC TAT CAG ACG AAA C 3′, *S. haematobium*—Reverse Primer: 5′ TCA CAA CGA TAC GAC CAA C 3′, *S. haematobium*—Probe: 5′ Joe-TGT TGG AAG ZGC CTG TTT CGC AA-BHQ1 3′ all synthesized by Biomers.net, Ulm, Germany. Primers and a probe were added for the detection of Phocid herpesvirus (PhHV) DNA as internal positive control (PhHV—Forward Primer: 5′ GGG CGA ATC ACA GAT TGA ATC 3′, PhHV—Reverse Primer: 5′ GCG GTT CCA AAC GTA CCA A 3′, PhHV—Probe: 5′ Cy5.5-TTT TTA TGT GTC CGC CAC CA-BBQ 3′).

The qPCRs were performed in a total reaction volume of 25 µl containing 12.5 µl of HotStarTaq Mastermix (Qiagen, Hilden, Germany), 1.75 × 10^−8^ mol MgCl_2_, 1.25 × 10^−13^ mol of each *Schistosoma* primer, 6.25 × 10^−14^ mol of the *Schistosoma* probes, 1.0 × 10^−12^ mol of the PhHV primer, 1.25 × 10^−12^ mol of the PhHV probe, 1.425 µg of the PhHV DNA template, 4 × 10^−5^ mg bovine serum albumin and 5 µl DNA eluate. In each run two positive controls (*S. mansoni* and *S. haematobium* DNA) and a negative control (H_2_O) were included. The qPCR was conducted using Corbett RotorGene 6000 (Qiagen, Redwood City, USA) with following steps: 15 min at 95 °C followed by 50 cycles of 15 s at 95 °C and 60 seconds at 60 °C with an initial touchdown from 65 to 60 °C in the first 11 cycles. The readout resulted from the RotorGene 6000 Software v.7.87 (Qiagen, Hilden, Germany). Results with a clean sigmoid curve within the PCR cycles were considered positive [39].

### Statistical analysis

All analyses were conducted using R^®^ v.4.1.2 (R Foundation for statistical computing, Vienna, Austria). Continuous variables were described using median and interquartile ranges (IQR). Categorical variables were presented using frequencies and percentages.

To estimate the prevalence of schistosome infection among the study population, relative frequencies of positive test results along with exact 95% confidence intervals (*CI*s) were determined.

For the risk factor analyses, the study population was divided into two distinct groups according to the areas of endemicity based on the species-specific PCR results. Test result distribution among individuals with various risk factors (study site, sex, age, education level, ever been treated with praziquantel, working as a farmer and being the main contributor to the family income) was described using frequencies and percentages. Univariable and multivariable logistic regression were performed to derive unadjusted and adjusted odds ratios (*OR*) and 95% *CI*s.

### Ethical consideration

Ethical clearance was obtained from the National Ethics Committee of Madagascar (ref. no. N°23-MSANP/CERBM, 05/03/2018) and the Ethics Committee of the Hamburg State Medical Chamber in Germany (ref. no. PV7019-4419-BO-ff, 29/10/2019).

All participants were informed about the aims of the study and its procedures in the local language (Malagasy). Study participation was voluntary and informed consent for the participation was obtained from the individual participant by signature or, in case of illiteracy, through a thumbprint in the presence of an independent witness. In all cases, participants had the right to refuse participation and withdraw the informed consent at any time without giving reasons. No monetary incentives were released to participate in the study and exclusively travel costs were reimbursed, calculated on the basis of the distance of the participants’ households from the PHCC.

## Results

### Study population

A total of 1482 adults were included in the study. Missing data are reported in the sections profession (0.8%) and praziquantel treatment (4.6%). All study sites were equally represented (ranging between 488 and 498 individuals across sites). The sex was balanced with highest proportion of females in Tsiroanomandidy (60.2%, Table 1), 54.5% females in Ankazomborona and 47.6% females in Andina.

**Table 1:** Study population characteristics

Characteristics	Andina *n* (%)	Tsiroanomandidy *n* (%)	Ankazomborona *n* (%)
Total	496 (33.5)	498 (33.6)	488 (32.9)
Female sex	236 (47.6)	300 (60.2)	266 (54.5)
Age (years)^1^	38.5 (27.0–50.0)	36.0 (25.0–49.8)	28.0 (21.0–40.0)
Age group (years)^1^			
18–29	155 (31.2)	181 (36.3)	255 (52.3)
30–39	101 (20.4)	92 (18.5)	97 (19.9)
40–49	104 (21.0)	100 (20.1)	68 (13.9)
50 +	136 (27.4)	125 (25.1)	68 (13.9)
Education level			
Secondary school or higher	134 (27.0)	280 (56.2)	211 (43.2)
Primary school	313 (63.1)	160 (32.1)	152 (31.2)
Never gone to school	49 (9.9)	58 (11.7)	125 (25.6)
Never treated with praziquantel	353 (71.2)	142 (30.9)	118 (25.8)
Working as a farmer	471 (95.0)	314 (63.1)	370 (76.4)
Main contribution to family income			
Yes	338 (68.1)	311 (62.4)	257 (52.7)
Of whom female	140 (41.4)	152 (48.9)	101 (39.3)
No	158 (31.9)	187 (37.6)	231 (47.3)
Of whom female	96 (60.8)	148 (79.1)	165 (71.4)

Stratified by study sites (*n* = 1482)

^1^Median age and interquartile range

Participants’ age ranged from 18 to 84 years. Lowest median age of 28 years was described in participants from Ankazomborona. The majority of participants were aged between 18 and 29 years across all sites.

Most of the participants reported an education level of secondary school or higher in Tsiroanomandidy and in Ankazomborona. In Andina the majority had only completed primary school.

More than two thirds of the population has never been treated with praziquantel in Andina while it was less than a third at the other two study sites.

Farming was the most common occupation at all sites. However, the distribution was not equal: in Andina almost everyone was a farmer, while there were less in Ankazomborona and rarely in Tsiroanomandidy.

At all sites most of participants were the main contributor to the family income. In the group of the main contributors a higher percentage were males, while a higher percentage of females stated that they were not the main contributor.

### Prevalence of schistosome infection in study area

Of importance, the *S. mansoni* infection showed dominant prevalence of 59.5% (95% *CI*: 55.0–63.8) in the rural area Andina and 50.2% (95% *CI*: 45.7–54.7) in the urban area Tsiroanomandidy in the central area of Madagascar and 1.6% (95% *CI*: 0.7–3.2) in peri-urban area Ankazomborona in the north-western part (Fig. 1). Mono-infections with *S. haematobium* were not found in Andina, while they were identified in 1.0% (95% *CI*: 0.3–2.3) of the participants in Tsiroanomandidy and 61.3% (95% *CI*: 56.8–65.6) in Ankazomborona. The prevalence of co-infections was generally low with 1.0% (95% *CI*: 0.3–2.3) in Andina, 0.6% (95% *CI*: 0.1–1.8) in Tsiroanomandidy and highest in Ankazomborona with 3.3% (95% *CI*: 1.9–5.3). Additional background information is listed in the Additional file 1: Table S1, specifying place of birth and residence of co-infected participants (*n* = 24). Endemicity of place of birth and place of home differed in six participants (25%), who were all participants in Ankazomborona.

### Risk factors for schistosome infection

**Fig. 2:**
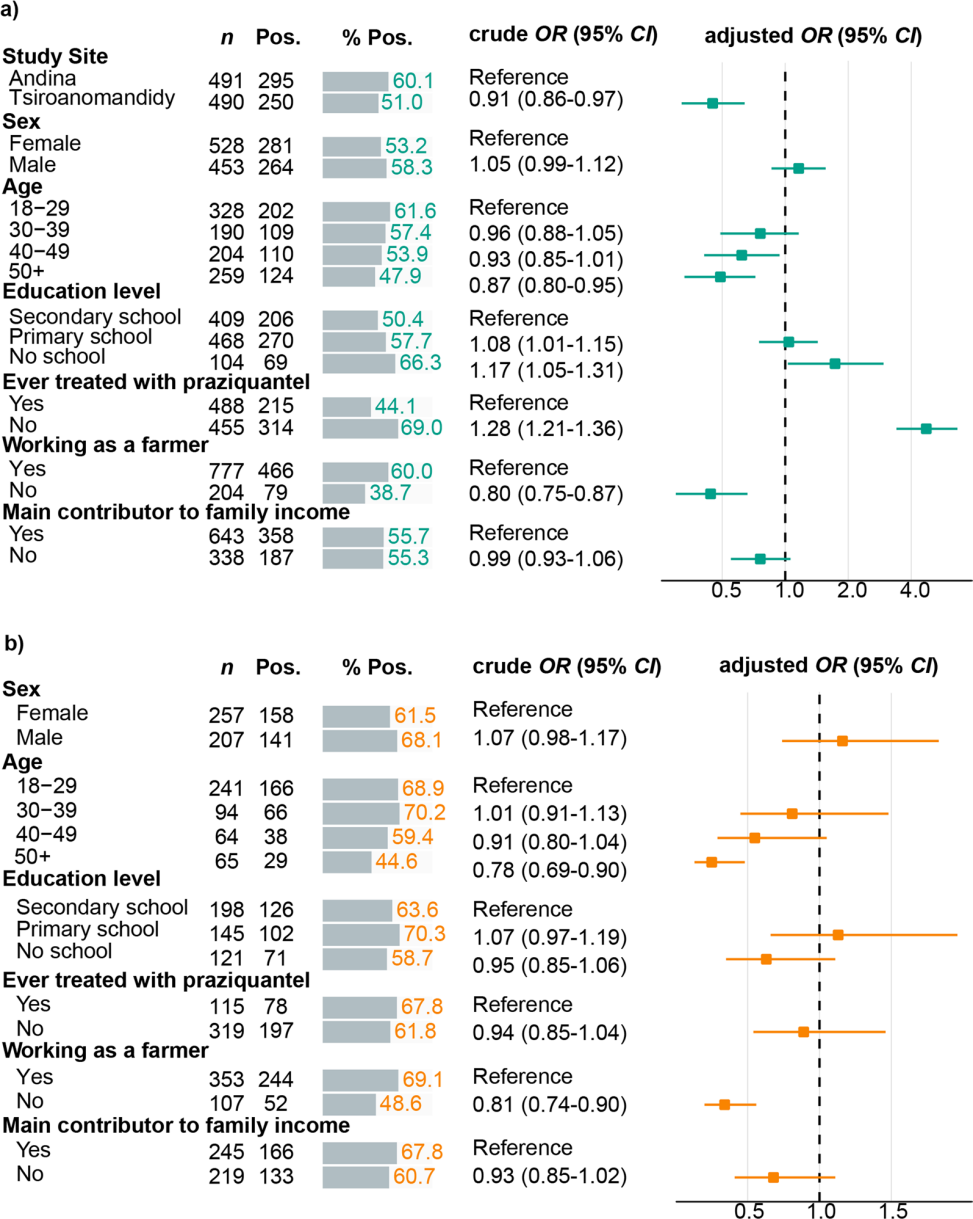
Risk factor analysis for PCR positivity of participants enrolled at (**a**) *Schistosoma mansoni* (*n* = 981) and (**b**) *S. haematobium* (*n* = 464) endemic study sites. Positivity rates, univariable and multivariable logistic regression with effect estimates in terms of crude and adjusted *ORs* and 95% *CI*. Variables that were controlled for in the multivariable regression, included: study site (when applicable), sex, age groups, education level, ever treated with praziquantel, working as a farmer and being main contributor to the family income. *CI* confidence interval*, n* sample size, *OR* odds ratio, *PCR* polymerase chain reaction, *Pos.* positivity frequencies*, % Pos.* positivity percentages

For the risk factor analysis co-infected participants (*n* = 24) and participants positive for the non-endemic species (*S. mansoni* area: *n* = 5, *S. haematobium* area: *n* = 8) were excluded yielding *n* = 981 participants from *S. mansoni* and *n* = 464 participants from *S. haematobium* endemic regions evaluable for risk factor assessments. Associations between risk factors and *S. mansoni* and *S. haematobium* infection status from logistic regression models are shown in Fig. 2. Adults attending the PHCC Tsiroanomandidy had lower odds of infection with *S. mansoni* when compared to adults attending the PHCC in Andina (adjusted *OR* = 0.45, 95% *CI*: 0.32–0.64). Higher infection rates of *S. mansoni* (adjusted *OR* = 1.16, 95% *CI*: 0.86–1.56) and *S. haematobium* (adjusted *OR* = 1.16, 95% *CI*: 0.74–1.83) were more likely to be observed in males than in females*.* Age groups older than 18–29 years, especially the 50 + age group, tended to have lower odds of infection with *S. mansoni* (adjusted *OR* = 0.49, 95% *CI*: 0.33–0.72) and *S. haematobium* (adjusted *OR* = 0.25, 95% *CI*: 0.13–0.48). Illiteracy increased the odds of infection with *S. mansoni* (adjusted *OR* = 1.73, 95% *CI*: 1.03–2.95) while infection rates with *S. haematobium* varied across education levels being lowest in participants with no school education (58.7%). Odds of infection also varied among both species regarding the previous treatment of praziquantel. Participants, who have never been treated with praziquantel before, tended to have higher odds of infection with *S. mansoni* (adjusted *OR* = 4.72, 95% *CI*: 3.40–6.65), while chances of infection with *S. haematobium* were lower (adjusted *OR* = 0.89, 95% *CI*: 0.54–1.46). If not working as a farmer participants had lower odds of infection with *S. mansoni* (adjusted *OR* = 0.44, 95% *CI*: 0.30–0.66) and *S. haematobium* (adjusted *OR* = 0.34, 95% *CI*: 0.20–0.56). Similar *S. mansoni* positivity rates were observed between being main contributor to the family income and not being main contributor to the family (adjusted *OR* = 0.76, 95% *CI*: 0.55–1.06). Odds of infection with *S. haematobium* were lower when not being main contributor to the family income (adjusted *OR* = 0.68, 95% *CI*: 0.41–1.11).

## Discussion

This cross-sectional study reports findings of elevated prevalence of schistosome infections in adults with *S. mansoni* in central and *S. haematobium* in north-west of Madagascar. Our data show an alarming gap for the accomplishment of UHC in endemic countries like Madagascar as adults are at risk of infection with schistosomiasis, but excluded by structured control programs for the disease.

To our knowledge this is the first study describing prevalence of both endemic species in Malagasy adults with a highly sensitive and specific serum PCR. With prevalence of around 60% in a *S. mansoni* and a *S. haematobium* endemic area we found similar prevalence as previous studies on different age groups in the country, indicating that beside school-aged children, also adults carry a high burden of infection in Madagascar. The geographic distribution of schistosome species here described, is also aligned with the current literature [31, 40] and seems to be associated with the distribution of the different species of freshwater snails [41] in the country.

Based on our findings, our study areas can be classified by WHO definition as high prevalence (> 50%) areas with need for interventions [10]. However, we show that less than half of the infected study population was never treated with praziquantel suggesting that they could have been a carrier of the infection for longer with a higher likelihood of developing the chronic form of the disease and serving as reservoir for the community [16]. Interestingly, in the *S. mansoni* endemic population, never having been treated with praziquantel, represents a risk factor for adult infections. Since it is extensively described that recovering from treatment does not represent a protective factor for further infections, we can speculate that exposure to treatment and/or MDA campaigns can raise awareness for the disease and induce less risky behaviors towards the infection. However, our findings show that treatment with praziquantel did not lower the odds of infection in *S. haematobium* areas. This supports the concept that many risk factors depend on the geographical and cultural contexts which need to be taken into account when planning interventions.

Our study provides new insights into the distribution of schistosomiasis among Malagasy adults, which will be essential for the shift of schistosomiasis control programs from targeted to broader groups of intervention as key element for the elimination of schistosomiasis and the promotion of UHC. As an example, the frequency of infection in our study population significantly decreased with increasing age, meaning that in these areas, control strategies should also target adults especially within the young working population of Madagascar. It is concerning to notice that young adults for whom productivity is supposed to be highest, are highly infested with a parasite notably known to weaken individuals productivity, creating disabilities and perpetuating the vicious cycle of poverty [7, 25, 42, 43].

Further, we observed higher prevalence of schistosome spp. in the rural (Andina) and peri-urban (Ankazomborona) areas than in the urban area (Tsiroanomandidy) and higher odds of infection with *S. mansoni* in Andina compared to Tsiroanomandidy. This is aligned with country data showing that rural populations of Madagascar are six times more likely to live without clean drinking water and twice as likely to have no access to sanitation in comparison to urban areas, increasing the risk to have contact with infested water [44]. Also, we observed a lower level of education in the rural area: while 56% of the participants in Tsiroanomandidy (urban) and 43% of the participants in Ankazomborona (peri-urban) reported a secondary school or higher education, only 27% of the participants in Andina (rural) had a secondary school or higher education. In the risk factor analysis we saw that a low level of education was associated with a higher frequency of schistosome infection in the *S. mansoni* group, which was previously explained by a higher likelihood of health illiteracy [45]. However, we could not observe the same in the *S. haematobium* group.

Very interestingly, our data show how two main characteristics identified as risk factors in the *S. mansoni* area (no school education and never been treated with praziquantel before) could not be described in the *S. haematobium* area. This might be due to the different cultural contexts, but in line with Wiegand, et al. [46], it can also be suggested that measurements for the two species should differ in order to prevent a neglect of diverting transmission and risk factors of the schistosome spp.

Interestingly, we also detected co-infections with the two endemic species and mono-infections with the non-endemic species (Figure 1). Even though the number of co-infected individuals was not particularly high, our data suggests that further investigations towards the migration history might reveal further risk factors of coinfections in Madagascar. This shows the importance also in diagnostics to promote tools that can allow the detection of both species with the same sample in order not to miss the presence of non-endemic species, which can further delay the diagnosis and treatment of schistosomiasis and lead to more complicated chronic forms of the disease especially in our global world, where mobility is becoming more frequent even in traditionally settled communities.

Previous control strategies by the WHO already recommend preventive treatment for adults with occupations, which bring them in steady contact with infested water, like farming, fishing and irrigation work [15] even though the alignment of national strategies is still delayed. Our data confirms the importance of occupational exposure as not working as a farmer, represented a strong protective factor for schistosome infection. As in Madagascar rice farming is the main occupation of the population [47], more tailored studies to deeply investigate the specific risk factors associated to farming are urgent in order to adapt infection control strategies. The higher prevalence of both species in men than in women could be attributed to the fact that in our study population men were more often the main contributors to the family income and thus engaging more in occupations like fishing or farming [48]. But in fact, in areas where farming or fishing is mostly done by women, higher prevalence of schistosomiasis was reported in women [42]. This shows that in shifting societies, occupational risk for schistosome infection should be addressed in a gender-neutral manner and accounting for the actual occupational risk of the specific communities.

This study has strengths and limitations. As a main strength, it assessed the schistosome infection status through a PCR methodology highly sensitive and specific allowing to distinguish the presence of two different schistosome species simultaneously [49–51]. Further, our study assessed the infection status of adults for the first time in rural, peri-urban and urban settings in Madagascar. The limitation of the applied methodology is that the test results may stay positive for a certain time following treatment [49, 52]. However, given that our sampling areas show a of high risk of transmission, we cannot exclude re-infections shortly after treatment. Moreover, the sampling approach through community workers did not guarantee the representativeness of the study population. Additional risk factors such as daily water activities, access to sanitation and hygiene behavior [53] could not have been explored in the frame of this study due to the structure of the investigation tool.

## Conclusions

Our study provides evidence of a high prevalence of schistosomiasis in adult populations of Madagascar. Diagnosis and treatment of the disease are a clear unmet medical need hampering the goal of schistosomiasis elimination as public health problem in the country by 2030. As our data show that prevalence of schistosomiasis can differ by schistosome species, geographic location, age, sex and other factors it requires targeted context specific, holistic and integrated control strategies to reduce morbidity and work towards guaranteeing the essential human right of health in all individuals. A shift in schistosomiasis control strategies is urgent to reinforce fragile health systems, positively impacting the fight against other diseases and health seeking behaviors of populations left behind from the UHC goal.

## Supplementary Information

**Additional file 1: Table S1.** Mobility history of co-infected participants.

## Data Availability

Data and materials can be made available upon specific request.

## Abbreviations

*CI*: Confidence interval
CRF: Case report form
DNA: Deoxyribonucleic acid
IQR: Interquartile range
MDA: Mass drug administration
NTD: Neglected tropical disease
*OR*: Odds ratio
PHCC: Primary health care centre
PhHV: Phocid Herpes virus
QALY: Quality-adjusted life-years
qPCR: Quantitative polymerase chain reaction
SDGs: Sustainable development goals
WASH: Water, sanitation and hygiene
WHO: World Health Organization
UHC: Universal health coverage
UN: United Nations
