# Supplementary material for: Prevalence and risk distribution of schistosomiasis among adults in Madagascar: a cross-sectional study
Source: Infect Dis Poverty. 2023 Apr 25;12:44. doi: 10.1186/s40249-023-01094-z (PMC10127445; doi:10.1186/s40249-023-01094-z)
Supplement: Supplementary file 1 — Additional file 1: Table S1. Mobility history of co-infected participants. [file 40249_2023_1094_MOESM1_ESM.pdf]

Supplementary Table 1. Mobility history of co-infected participants. In orange: *S. haematobium* endemic, in green: *S. mansoni* endemic

Study Site	Place of birth	Place of home
**Ankazomborona (north-western)**	Fianarantsoa	Ambondromamy
	Ambararata fotsy	Ambonara
	Ambohimahasoa	Amboromalandy kely
	Ambositra	Ambonara
	Ankazomborona	Amboromalandy kely
	Ankazomborona	Madiromiongana
	Ankazomborona	Madirovalo
	Bekarara Ambony	Amboromalandy
	Fianarantsoa	Ambondromamy
	Fianarantsoa	Ambormalandy kely
	Fianarantsoa	Amboromalandy kely
	Madiromiongana	Madiromiongana
	Madiromiongana	Madiromiongana
	Maromanihy	Ambonara
	Marovoay	Madirovalo
	Tsaravotra Tsaratanana	Amboromalandy
**Tsiroanomandidy (central)**	Belohitsiribihina	Tsaratanàna
	Tsaratanàna	Tsaratanàna
	Tsiroanomandidy	Androtra
**Andina (central)**	Andina	Ampasina
	Andina	Ampasina
	Andina	Ampasina
	Andina	Ampasina
	Andina	Ampasina
